# Unlocking renewable energy potential: Overcoming knowledge sharing hurdles in rural EU regions on example of poland, sweden and france

**DOI:** 10.1371/journal.pone.0320965

**Published:** 2025-04-10

**Authors:** Justyna Żywiołek, Radosław Wolniak, Wieslaw Wes Grebski, Sunil Tiwari, Marek Matuszewski, Adam Koliński

**Affiliations:** 1 Faculty of Management, Czestochowa University of Technology, Czestochowa, Poland; 2 Silesian University of Technology, Gliwice, Poland; 3 Penn State Hazleton, Pennsylvania State University, 76 University Drive, Hazleton, Pennsylvania, United States of America; 4 Department of Tourism Studies, School of Business Studies, Central University of Kerala, Periye, Kerala, India; 5 Department of Convergence Management, Korea University, Seoul, South Korea; 6 Poznan School of Logistics, Poznan, Poland; 7 Lukasiewicz Research Center - Poznan Institute of Technology, Poznań, Poland; University of Coimbra: Universidade de Coimbra, PORTUGAL

## Abstract

The optimal technological choice for sustainable development lies in renewable energy sources (RES). However, the potential offered by RES utilization poses significant challenges for mobile technologies and everyday living. Despite extensive research and information highlighting the benefits of renewable energy, there remains considerable debate, and limited awareness persists. The advantages of RES are not fully comprehended, raising concerns about its consistent application. Regrettably, lack of knowledge and a fundamental understanding hinders effective dissemination. To gauge the attitudes of residents in regions where RES is employed, this study employed a questionnaire authored by the researcher. The study was conducted between June 2022 and January 2023, with a total of 12,428 participants completing the survey. The sampling method utilized an online form distributed via various social media channels and among local contacts of the authors in Poland, Sweden, and France. Gender allocation: 58% male and 42% female. Respondents shared their perspectives on ecology and disclosed their familiarity with RES utilization. Results indicate public skepticism regarding the adequacy of RES security measures and the level of knowledge for its effective use. Insufficient experts, limited social advocacy, and reliance on online sources contribute to a low level of awareness. In several EU countries, the absence of widely accepted and easily accessible information on renewable energy sources (RES) hinders knowledge sharing and adoption. Despite the EU’s efforts to promote renewable energy through directives and subsidies, rural communities in these countries often lack adequate education and awareness about RES technologies. This gap in knowledge contributes to unfavorable perceptions, with some residents viewing renewables as unreliable or economically unfeasible options compared to traditional energy sources like coal or natural gas. Additionally, bureaucratic hurdles and inconsistent government policies further complicate the transition to renewable energy, discouraging investment and innovation in the sector. As a result, while the EU aims for a sustainable energy future, these barriers impede the widespread growth of RES and hinder progress towards climate targets. In Poland the study found that 76% of respondents expressed favorable perceptions of RES, indicating a general inclination towards adopting clean energy solutions. In Sweden, the analysis uncovered a high level of environmental awareness among participants, with 85% of respondents expressing concern about environmental degradation. Despite this awareness, 62% of participants reported reservations about the security and affordability of energy derived from renewable sources. Additionally, 48% of respondents expressed uncertainty or ambivalence regarding the environmental benefits of RES. In France, the research revealed similar concerns among respondents regarding the security and affordability of renewable energy. 59% of participants expressed reservations about the security of energy derived from renewable sources, while 53% cited perceived high costs as a barrier to adoption. Furthermore, 41% of respondents identified underdeveloped RES infrastructure as a hindrance to wider acceptance and utilization. The quantitative data highlights the complex landscape of renewable energy perceptions and attitudes in Poland, Sweden, and France. While there is a general awareness of environmental issues and a positive inclination towards clean energy solutions, concerns about security, affordability, and infrastructure remain significant barriers to widespread adoption. These findings underscore the importance of targeted interventions and educational efforts to address these challenges and promote sustainable energy practices across Europe. Renewable energy sources (RES) represent a critical avenue for sustainable development, offering a pathway to mitigate environmental degradation and reduce dependence on fossil fuels. This study investigates public attitudes, knowledge levels, and barriers to RES adoption in rural areas of Poland, Sweden, and France, highlighting the unique socio-economic and cultural factors influencing these regions. Conducted between June 2022 and January 2023, the research utilized an online survey, gathering responses from 12,428 participants across these countries. Respondents evaluated statements on environmental responsibility, RES knowledge and application, and perceived obstacles, using a five-point Likert scale. Key findings reveal that while environmental awareness is high, significant barriers persist in the form of limited knowledge, underdeveloped infrastructure, and perceptions of high costs associated with RES. In Poland, 76% of respondents expressed a positive view of RES but cited concerns about cost and security. Swedish participants demonstrated strong environmental awareness (85%), yet 62% voiced reservations about RES affordability and reliability. French respondents similarly highlighted concerns regarding infrastructure and costs, with 41% identifying underdeveloped RES systems as a primary hindrance. The study underscores the importance of targeted educational campaigns and policy interventions to bridge knowledge gaps and foster greater acceptance of RES. Tailored strategies addressing local barriers—such as financial incentives, community-based advocacy, and infrastructure investments—are essential to overcoming these challenges. By exploring diverse perspectives and barriers across the three countries, this research contributes valuable insights to the broader discourse on sustainable energy transitions in the EU.

## 1. Introduction

Political and economic upheavals globally have had significant impacts on many countries, especially inside the European Union (EU), sparking a strong interest in renewable energy sources (RES) technologies [1]. The increased interest is driven by the acknowledgment of the critical need for sustainable energy solutions amidst immediate environmental issues and geopolitical risks. The EU has become a central hub for renewable energy efforts, implementing various policies and legal modifications to promote the use of renewable energy sources and guide member states towards a more environmentally friendly and sustainable future [2]. The EU has established ambitious goals to increase its renewable energy capacity and decrease carbon emissions, indicating a significant change towards a more sustainable energy environment [3]. The goals consist of reaching at least 40% renewable energy in the overall energy mix by 2030, reducing greenhouse gas emissions to 55% below 1990 levels, and aiming for climate neutrality by 2050 [4–6]. The EU is dedicated to promoting sustainable development and reducing dependence on imported energy sources, making the energy transition a key aspect of its climate action agenda [7]. Advancements in renewable energy technology, particularly solar panels and heat pumps, are crucial in this transition to a new energy paradigm [8]. Solar photovoltaic (PV) energy has become more popular in the EU due to its cost-effectiveness, eco-friendliness, and adaptability [9]. The EU is rapidly increasing its solar energy capacity, projected to reach 158 GW by the end of 2021, highlighting the region’s shift towards solar energy as a feasible substitute for conventional fossil fuels. Heat pump technology has advanced significantly in improving energy efficiency and decreasing carbon emissions to meet the heating and cooling requirements of buildings across the EU [10]. Recent political and economic disruptions worldwide, primarily affecting European Union (EU) nations, have sparked a significant surge in interest in renewable energy sources (RES) technology. The multitude of initiatives, resources, and policies underscore the critical importance of advancing RES installation technology within the EU [11]. Recent legislative changes in the EU signal ambitious targets, such as achieving a minimum of 40% renewable energy in the total energy mix by 2030, reducing greenhouse gas emissions to 55% below 1990 levels, and attaining climate neutrality by 2050 [12]. These measures are expected to play a significant role in promoting sustainable development within EU countries and diminishing dependence on energy imports. The EU has prioritized the energy transition as a key strategic focus in its efforts to combat climate change and enhance energy security [13]. With the continuous advancement and increasing prevalence of renewable energy technologies, their impact is projected to become even more profound [14,15].

On May 18, 2022, the EU unveiled its new external energy strategy, outlining the path toward green energy transition, heightened energy security, and diversification of energy supplies [16,17]. The ongoing global energy crisis has spurred EU countries to intensify efforts to attain energy independence from Russia [18,19]. However, the approaches adopted in individual countries’ energy policies vary significantly due to differences in their energy mix structures and supply sources [20–22]. This study focuses on Poland, Sweden and France to assess the impact of the so-called energy crisis on the short-term energy security strategies of EU countries.

The selection and comparison of Poland, Sweden, and France in this study offer a nuanced exploration of public attitudes and perceptions towards renewable energy sources (RES) within diverse socio-economic and geographical contexts [23]. Poland represents a nation grappling with the transition from coal-dominated energy to renewables, reflecting the challenges faced by countries heavily reliant on fossil fuels. Sweden’s progressive renewable energy policies and extensive use of hydropower and wind energy provide insights into successful adoption strategies. France’s unique energy landscape, characterized by a significant nuclear energy capacity alongside growing interest in renewables, offers a contrasting perspective [24]. By analyzing these countries, the study can capture a broad spectrum of attitudes, challenges, and best practices related to RES adoption, contributing to a more comprehensive understanding of global energy transitions.

In Poland, the study focuses on rural areas such as Podlaskie and Lubusz, characterized by expansive agricultural landscapes and ample sunlight [25,26]. These regions are ideal for solar photovoltaic (PV) installations, offering opportunities for both residential and agricultural applications. Additionally, biomass heating systems hold promise due to the availability of agricultural residues and forest biomass, particularly in regions with strong agricultural activity [27]. In Sweden, attention is directed towards rural regions such as Dalarna and Västra Götaland, known for their diverse landscapes ranging from forests to coastal areas [28]. These areas have significant potential for wind energy generation, especially offshore wind farms along the coastlines. Moreover, biomass heating systems utilizing forestry residues and organic waste could be viable options for meeting heating needs in these regions [29,30]. In France, the study targets rural areas such as Occitanie and Nouvelle-Aquitaine, characterized by varied landscapes including mountains, forests, and agricultural plains [31,32]. These regions offer favorable conditions for solar PV installations, given the abundant sunlight throughout the year. Additionally, biomass energy production using agricultural residues and forestry biomass could play a crucial role in meeting energy demands, particularly in regions with strong agricultural and forestry sectors [33,34].

The primary objective of the study is to analyze and evaluate how selected countries respond to changes in renewable energy implementation in households and businesses. The focus is on understanding their level of knowledge and identifying the obstacles they encounter. It is essential to clarify that the intention is not to evaluate energy policy; instead, the aim is to highlight countries that proactively identify and address barriers. The primary goal of this study was to pinpoint areas where perspective respondents’ views towards knowledge-sharing barriers about renewable energy sources needed to be improved. The following are the key findings from the research that has been presented:

To achieve this, the study poses the following research questions:

1) What tools can countries use to remove barriers to sharing RES knowledge?
2) How did the countries deal with the negative consequences of a lack of national action in the field of RES knowledge?
3) What are the key factors influencing public acceptance of renewable energy projects in different contexts?
4) How can governments address resistance from communities dependent on traditional energy industries to foster greater social acceptance of renewable energy solutions?

## 2. Literature background

### 2.1. European clean energy policy

A multi-year roadmap for decarbonizing key economic sectors was issued by the European Commission. The achievement of climate neutrality is the major objective of this approach. Additionally, it paves the way for the creation of hydrogen technologies, which guarantee energy efficiency and consume less resources. The EU wants to encourage the production of hydrogen and the growth of renewable energy sources (RES) [35–37]. A proclamation encouraging collaboration in the area of hydrogen technology has the support of 28 different European nations. One hundred businesses, institutions, and organisations are involved in this collaboration. It is important to note that a growing number of nations view hydrogen as the fuel of the future, which is essential for the decarbonization of the transportation sector.

**Fig. 1:**
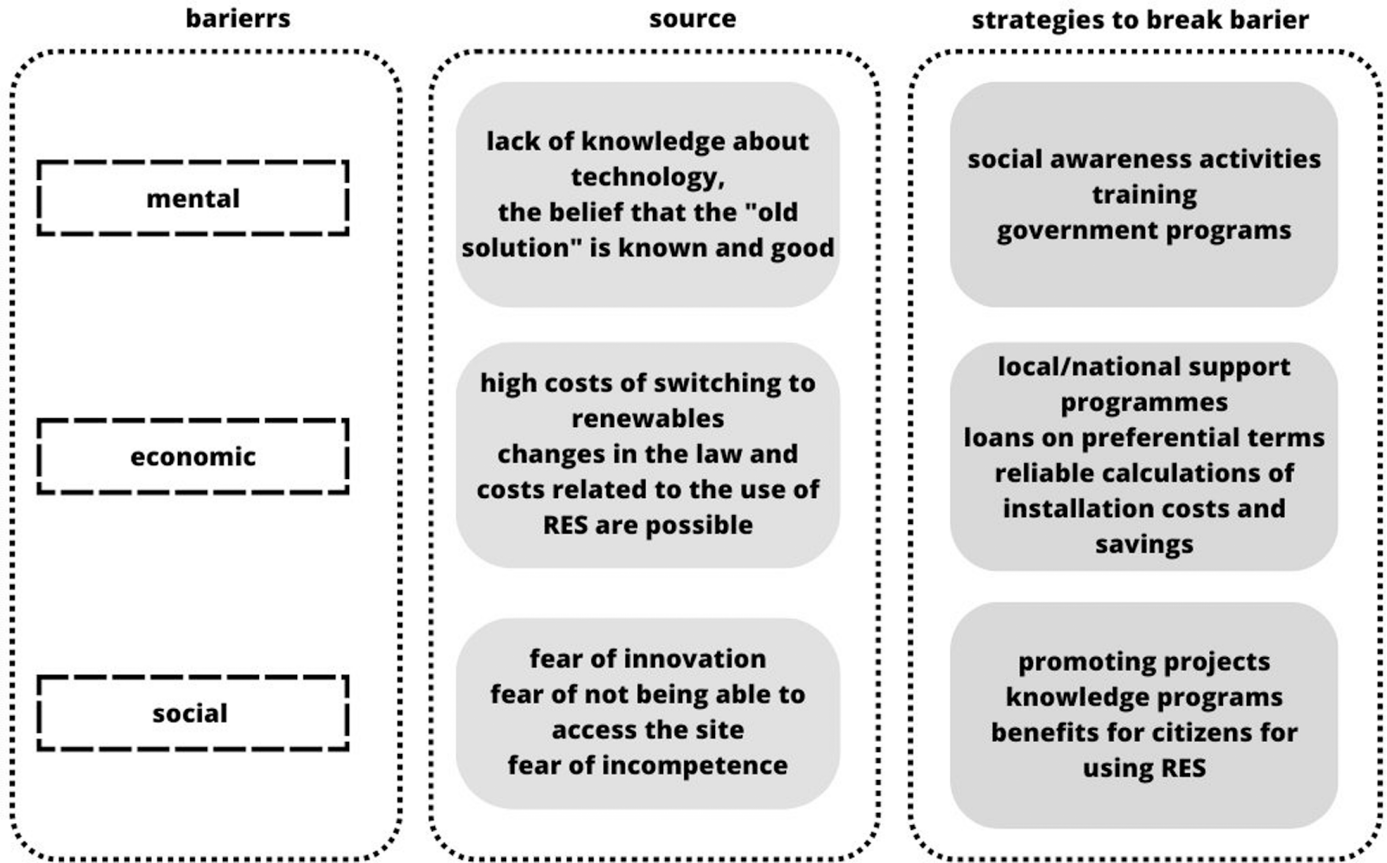
Identified social, economic and mental barriers to the use of renewable energy in the surveyed countries.

How can social responsibility and awareness be used to persuade society to utilise renewable energy sources? Consumers are frequently informed that renewable energy is expensive by several sources of information. To overcome society’s mental, social, and economic hurdles is therefore important for the development plan (Fig. 1). The figure 1 illustrates the key barriers to the adoption of renewable energy sources (RES), their origins, and strategies to overcome them. This presentation aims to organize information about the challenges faced by society and the economy in the context of the energy transition and to indicate possible directions for actions supporting the development of RES. The diagram serves as a visual introduction to the topic of barriers and solutions that can accelerate the process of transitioning to more sustainable forms of energy.

The fig 1 illustrates the key barriers to renewable energy adoption, categorized as mental, economic, and social, alongside their respective sources and strategies to address them. It highlights that addressing these barriers requires targeted interventions, such as awareness programs for mental barriers, financial support for economic challenges, and community-focused initiatives for social resistance. In analyzing the barriers to renewable energy adoption in Poland, Sweden, and France, this framework provides a foundation for understanding how specific obstacles manifest in different national contexts. Each country’s unique energy infrastructure, economic dependencies, and societal attitudes necessitate tailored strategies to effectively address these challenges and foster the transition to sustainable energy systems.

The energy transition towards renewable energy sources is a multifaceted process that encounters various barriers arising from local economic, social, and mental conditions. An analysis of Poland, Sweden, and France demonstrates how specific historical, cultural, and structural factors influence the pace and scope of change in these countries, highlighting the complexity of the challenges associated with shifting to more sustainable energy sources. In Poland, the primary obstacle to transformation is the country’s heavy reliance on coal, which has served as the foundation of its economy and energy security for decades. The extensive coal infrastructure and economic ties of the mining industry to numerous regions make the transition to renewable energy sources a significant financial challenge. Coal mines are not only a source of income for thousands of workers but also a vital part of the cultural identity of mining regions. As a result, changes in the energy structure are often perceived as a threat to jobs and local communities. Skepticism towards renewable energy is further reinforced by concerns about its reliability and costs, making it more difficult to build trust in new technologies. In Sweden, the situation is markedly different—the country is a global leader in renewable energy development, with a high level of environmental awareness among its population. Thanks to its long-standing commitment to innovation and a stable economy, Sweden has successfully advanced technologies such as wind, solar, and biomass energy. Nevertheless, despite widespread public support, challenges remain. High initial investment costs and the need to modernize the transmission infrastructure represent a considerable financial burden, even for a developed country like Sweden. Local opposition to projects such as wind farms often stems from concerns about landscapes and potential environmental impacts, which paradoxically reflect the society’s deep ecological awareness. France, where the energy sector is heavily dominated by nuclear power, faces unique challenges. The dominance of nuclear energy, seen as a symbol of stability and low emissions, generates both benefits and obstacles. Low electricity prices, resulting from the high efficiency of nuclear plants, provide a competitive advantage but also make it harder for renewable energy sources to compete with the well-established system. Moreover, issues related to reactor safety, nuclear waste management, and the need to modernize aging installations create new economic challenges. At a social and mental level, nuclear energy is perceived as a reliable and scalable solution, which hinders the development of alternative technologies. However, growing environmental awareness and concerns about nuclear safety are beginning to drive interest in renewable energy sources. Mental barriers are a particularly important, though often underestimated, aspect of the energy transition. In Poland, many people perceive renewable energy as expensive and unstable, further strengthening attachment to traditional solutions such as coal. Similarly, in Sweden, despite general acceptance of the green transition, concerns arise about the variability of certain energy sources, such as wind power, and their impact on the aesthetics of the landscape. In France, mental barriers are linked to attachment to the stability of nuclear energy and doubts about the ability of renewables to provide long-term energy security. The analysis of Poland, Sweden, and France illustrates that the energy transition requires not only advanced technologies but also a comprehensive approach that takes into account local economic, social, and psychological conditions. Poland needs strategies that allow for a gradual shift away from coal while supporting mining regions and building trust in new technologies. Sweden, despite its achievements, must address local conflicts and high investment costs, which require further commitment to innovation and dialogue with local communities. France, on the other hand, faces the challenge of balancing its strong nuclear traditions with the growing need to develop renewable energy sources. These three cases emphasize the complexity of energy transition processes, which demand integrated actions at political, economic, and social levels. Only by effectively managing diverse barriers, both tangible and intangible, can the foundation for a more sustainable energy future be established. This transition requires not only investments in technology but also the building of public trust, education, and open dialogue that considers the diverse perspectives and needs of each country. In summary, the selection of Poland, Sweden, and France in the study allows for an examination of diverse economic, social, and mental barriers to renewable energy adoption, offering insights into the complexities of energy transitions within different national contexts.

Lack of knowledge about energy sources that are renewable and the possibility of receiving energy from sources other than fossil fuels creates mental obstacles, sometimes even from a complete lack of understanding. Second, the barrier (social barrier) is based on societal norms that include familiarity with established technology and a perception of unpredictability and danger from emerging (rarely used) technologies [44]. Third economic barrier is a logical result of the economic circumstances of the populace [45–47]. In highly advanced and prosperous communities, it is significantly simpler to overcome this obstacle [48–51]. However, because it requires enormous amounts of start-up money from the populace, it is particularly challenging for poor countries to overcome [52]. For an accurate evaluation of contrasting technologies in terms of their benefits to society and the economy, a greater level of knowledge is also required [53–55]. Since it was believed that the typical person knows nothing about the manufacturing, storage, and transportation of hydrogen, the technological barrier was left out of Fig 1. In contrast, studies on technology transfer and the so-called commercial usage of technology should take the technological barrier into consideration [56]. Most energy technology implementation projects are evaluated using sustainability-based criteria and the concept of corporate social responsibility [57,58]. The engagement and contribution (influence) of businesses to sustainable development is the broad definition of CSR [59]. It is a method for incorporating the company’s principles into the culture and for making choices, plans, and actions in an open and accountable fashion, i.e., by voluntarily improving the social situation and the environment. Increasing social responsibility is only achievable, according to the research [60,61], through more transparency on social and environmental issues using plainer language. Only by raising public awareness through numerous educational and cognitive campaigns, social campaigns, the implementation of hydrogen energy promotion projects, and the introduction of subsidies and incentives to support the green energy industry can barriers be broken down, resulting in rebates, profits, and financial relief [62]. The calculations used in the real economy presentation relate to the energy and money balances. Designing a promotional strategy should thus concentrate on the most crucial (readable) aspects, such as the vehicle’s overall cost and its price fuel efficiency [63,64], or long-term fuel economy [65–68]Financial incentives, a robust and convenient charging infrastructure, and the presence of manufacturing facilities in the vicinity may all be contributing factors to the expansion of the electric vehicle market in a given country [69]. Women usually view the usage of hydrogen energy more favourably than men do, Cherryman et al. found [42], with the main issues being cost and safety.

The knowledge management and generation system is a complicated system of associated components for performing fundamental knowledge functions [70]. These processes involve data gathering, processing, sharing, and analysis [71]. Humans build the knowledge management system [25,72], but it’s also crucial to consider the culture in which these structures and activities are carried out [73–75]. People produce and consume knowledge [76]. They determine the structure and operation of the system [77], as well as the efficacy and efficiency of its continuous operations, such as knowledge generation and exchange [78,79]. This is knowledge’s function in daily life, whether it is at home, at work, or in the community [80]. This is a direct effect of the value and function of knowledge in human existence. Confidence is the cornerstone of good and efficient communication [81]. There are two groups of technical and operational elements. From the perspective that possibilities form the system, this distinction is crucial. A crucial role is played by technical components, architecture related to human behaviour as well as applications and IT systems, such as management and relationship-building techniques [82,83]. However, much of this infrastructure is more delicate and subject to greater disruptions, which can either increase or decrease the degree of confidence [71,84,85]. The usage of alternative energy sources has also evolved into a national policy concern [86–88]. Despite the epidemic, the need for energy is still growing because to economic and technical advancement [89]. Numerous technologies that make it easier to run the home are a result of increased technology usage in the home, but they also result in an appreciable rise in energy consumption [90]. That’s why it is essential to manage habits that conserve energy. The two factors that most frequently cause this behaviour are financial savings and a commitment to sustainable development. It is vital to educate the customer about using these solutions and to increase his confidence in these energy sources in order to realise the significance of these variables [91,92]. Numerous ideas have been created by scientists to explain individual behaviour [93]. Prior research on behaviour and awareness in the area of energy savings was mostly focused on design scale and studies that examined the relationship between these two aspects [94]. The link between the adoption of alternative energy sources and psychological obstacles and challenges in spreading information about renewables is examined in this article [95]. One of the key element of the knowledge management process, which is based on a plan assuming the use of knowledge as a strategic resource [42], is information sharing. Without information sharing, there cannot be knowledge management [71,96]. To feel motivated to share information, one must believe that doing so would not jeopardise their ability to retain gained knowledge as a source of competitive advantage. As a result, it’s important to provide the right surroundings and circumstances for information transfer. The quality of interpersonal interactions and reciprocal trust, caring, and emotional engagement are given particular focus in the literature on the subject [97]. As a result, for the time being, the behaviour may be researched and examined in a particular setting to ascertain its defining state [89,98]. The project of examining client awareness and comprehension is long-term and involves a number of societal factors and resources, as well as assets provided by companies offering creative solutions [99] aims to pique consumer interest and trust [100,101].

Understanding and disseminating knowledge about Renewable Energy Sources (RES) holds paramount importance in fostering a sustainable and environmentally conscious society [102]. The study’s findings underscore the significance of awareness and comprehension of RES among the public, as it plays a pivotal role in shaping attitudes, behaviours, and, consequently, the broader adoption of renewable energy practices [103]. Also a nuanced understanding of RES contributes to the removal of barriers hindering their widespread adoption. Some studies [104] identifies concerns related to perceived high costs, underdeveloped infrastructure, and knowledge gaps. Addressing these concerns requires targeted initiatives that not only make renewable energy more economically feasible but also enhance public awareness about the benefits and safety of RES [105]. In a global context, nations equipped with comprehensive knowledge about RES gain a competitive edge in the race towards a sustainable future [106]. They can strategically invest in and harness renewable energy technologies, fostering economic growth while reducing their ecological footprint. This knowledge also plays a pivotal role in international collaborations and agreements, shaping the collective efforts to address climate change and promote a greener planet [107].On a societal level, education about RES fosters innovation and technological advancements. It fuels research and development initiatives, leading to breakthroughs in renewable energy technologies [108]. The dissemination of this knowledge encourages the emergence of a skilled workforce capable of driving the renewable energy sector forward, creating job opportunities and supporting economic diversification [109].

It can be stated that, understanding RES is crucial for energy security. By diversifying energy sources and reducing dependence on finite fossil fuels, nations can enhance their resilience to geopolitical uncertainties and fluctuations in global energy markets. This knowledge provides a foundation for building robust and sustainable energy systems that can withstand the challenges of the 21st century. The writers realised the need of focusing on two topics after conducting the necessary scientific observations. The first, and maybe more challenging, is understanding how to manage and use energy resources. The second is an openness to exchanging culturally relevant knowledge, particularly in connection to alternative energy sources [110,111]. In the table 1 there is a description of main factors of the impact of knowledge on the dissemination of Renewable Energy Sources (RES).

**Table 1:** The impact of knowledge on the dissemination of renewable Energy Resources (RES).

Factors	References	Description
Education and Awareness	Smith et al., Johnson, Lee et al., Wang, Martinez et al., Brown, Taylor	The level of education and awareness in a society directly influences the adoption of RES. Informed individuals are more likely to support and demand renewable energy solutions.
Policy and Regulations [	Garcia et al., Patel, Robinson et al., Harris	Government policies and regulations play a crucial role in shaping the energy landscape. Supportive policies, incentives, and regulatory frameworks can accelerate the integration of RES into mainstream energy systems.
Technological Advancements	Miller, Kumar et al., Chen, Nguyen	The pace of technological advancements in the field of renewable energy impacts its accessibility and efficiency. Breakthroughs in technology make RES more viable and attractive alternatives.
Investment and Funding	Anderson et al., Lopez, Kim et al., Thompson	Adequate financial support and investments are essential for the development and implementation of renewable energy projects. Funding availability can significantly impact the scale and speed of RES dissemination.
Public Perception and Acceptance	Davis, Martin, Wilson et al., Jackson	Public perception and acceptance of renewable energy influence its widespread adoption. Positive attitudes, cultural norms, and social acceptance contribute to the success of RES initiatives.
Infrastructure Development	White et al., Thomas, Hernandez et al., Scott	The availability and development of infrastructure, such as grid systems and storage solutions, play a vital role in the integration of renewable energy into existing energy networks. Adequate infrastructure supports RES dissemination.
Research and Development	Moore, Evans et al., Gonzalez, Perez	Ongoing research and development efforts contribute to the improvement of renewable energy technologies. Scientific advancements drive innovation, efficiency gains, and cost reductions, making RES more competitive.

Source: Authors own work on basis of [35–65].

The literature study [53,103,112–114] emphasises the crucial significance of comprehending and spreading information about Renewable Energy Sources (RES) to promote sustainable and ecologically aware communities. Three main obstacles to the general acceptance of RES are lack of understanding, social norms, and economic restraints. These obstacles appear in several ways, such as a lack of comprehension about renewable energy technology, cultural opposition stemming from experience with existing technologies, and economic difficulties, especially in less affluent countries.

To overcome these obstacles, specific programmes must be implemented to increase public knowledge, make renewable energy financially viable, and promote innovation and technical progress [115]. A comprehensive knowledge of renewable energy sources (RES) provides nations with a competitive edge in the global sustainability efforts, while also promoting economic growth, job creation, and energy stability [116]. Furthermore, it plays a crucial role in international collaborations and agreements focused on collectively addressing climate change [117].

The report highlights the importance of education in fostering innovation, study, and creation in the renewable energy industry. To establish robust and long-lasting energy systems, societies must provide individuals with necessary knowledge and skills to handle geopolitical instability and fluctuations in the global energy market [53].

Smith et al. [15] highlight the crucial role of cultural exchange and openness in facilitating the adoption of renewable energy, particularly in regions heavily reliant on traditional energy sources. By fostering a collaborative environment and promoting knowledge-sharing initiatives, organizations can accelerate the transition to renewable energy while mitigating the adverse effects of environmental degradation. Comprehending and spreading knowledge about renewable energy sources (RES) is crucial for attaining energy security, promoting economic development, and addressing environmental issues [118]. To ensure a sustainable future for future generations, societies must address impediments and raise awareness [55].

Challenges to Energy Security in the European Union: A Theoretical Framework Energy security can be defined in a variety of ways, ranging from physical supply interruptions to economic, environmental, and political factors [119,120]. The political repercussions of energy market changes [121,122]Energy security is described by the United Nations as “the ongoing accessibility of various forms of energy in sufficient quantities and at competitive costs.” [123,124]. Similar terminology is used by the International Energy Agency (IEA), which describes it as “the continuous availability of affordable energy resources [7,125–127].” But in the most general sense, “energy security” refers to a range of issues including the effects of climate change, globalisation, the uncertain future of fossil fuels, sustainability, energy efficiency, mitigating the release of greenhouse gases, availability to energy-related services, and energy insecurity [117,128].

The writers realized the need of focusing on two topics after conducting the necessary scientific observations [129]. The first, and maybe more challenging, is understanding how to manage and use energy resources. The second is an openness to exchanging culturally relevant knowledge, particularly in connection to alternative energy sources [110,111]. The following research questions were put out by the authors in combination with their observations, and the ensuing observations were reflected in the set of study objectives and hypotheses:

(H1). The level of knowledge about RES increases the level of willingness to use these energy sources

(H2). Barriers to the use of RES result directly from the level of knowledge about them.

## 3. Materials and methods

The study’s major goal was to identify the obstacles that regular people believed prevented them from learning more about RES. Participants in the study were residents of Poland, Sweden, and France, three EU nations. These are nations where the use of RES varies, hence the amount of citizen knowledge should also vary.

The survey’s design included a review of the literature, in particular, research of a comparable nature carried out in other nations, with a focus on the EU. A preliminary survey was carried out between June 2022 and January 2023 using an online form. The survey was developed based on a thorough literature review to ensure consistency with previous research on renewable energy sources (RES) and their social implications. The questionnaire was divided into three sections to address key research objectives: environmental responsibility, understanding and use of RES, and perceived barriers to their adoption. Each section consisted of five statements rated on a five-point Likert scale from 1 (strongly disagree) to 5 (strongly agree), reflecting a wide range of attitudes, knowledge levels, and concerns about RES. To ensure the reliability and validity of the instrument, a pilot test was conducted with a smaller, representative sample. This step was crucial to identify and resolve ambiguities in the wording and structure of the questions. The pilot testing process also verified the accessibility of the questionnaire to respondents with different educational and professional backgrounds, ensuring that the language and content were appropriate for a general audience. These refinements were designed to minimize potential misinterpretations and improve the quality of the collected data. The final survey was distributed online via social media platforms and local networks between June 2022 and January 2023 to respondents from Poland, Sweden, and France. A total of 12,428 responses were collected, providing a large data set for analysis. To verify the internal consistency of the survey, Cronbach’s alpha coefficients were calculated for each section, all exceeding 0.8, confirming a high degree of reliability.

**Fig 2:**
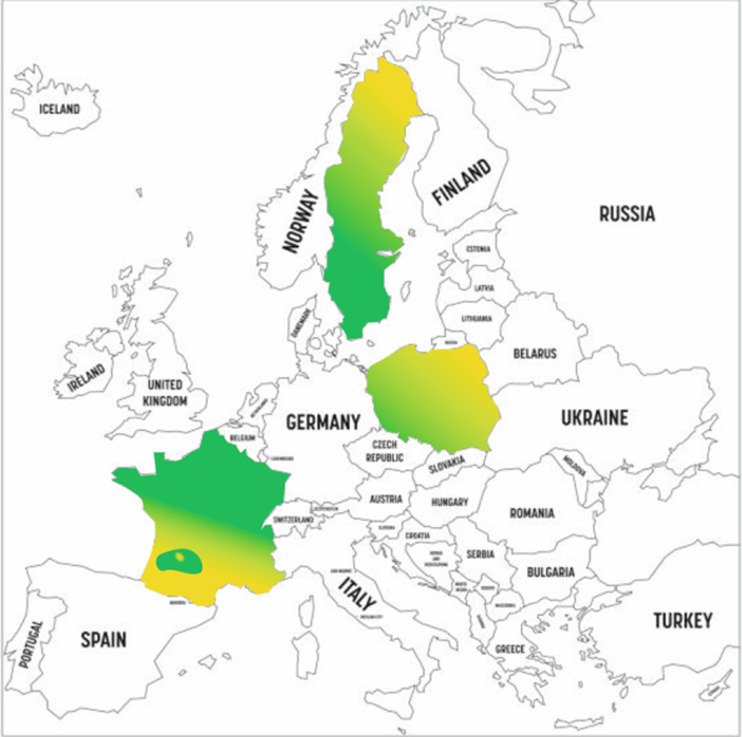
Geographical context of the study areas: Poland, Sweden and France. The map highlights the study regions, emphasizing the socio-economic diversity of the countries studied. (yellow indicates that respondents were reluctant to participate in the study, green indicates that less than 1% withdrew from the survey. Source: own study.

Understanding the geographical context of the study is crucial for interpreting the socio-economic and infrastructural diversity of the surveyed regions. The included map highlights the study areas in Poland, Sweden, and France, offering a visual representation of the countries chosen for their varying levels of renewable energy adoption and development. These regions were selected to capture a broad range of public perceptions, shaped by differences in energy infrastructure, policy, and cultural attitudes. By providing a clear illustration of the study’s scope, the map enhances the understanding of regional variations that influence societal attitudes toward renewable energy sources (RES) and the barriers to their adoption. The study was conducted in Poland, Sweden, and France, representing diverse socio-economic and energy landscapes (Fig 2).

The figure illustrates the geographical context of the study, highlighting the inclusion of Poland, Sweden, and France. These countries are depicted to represent the socio-economic and infrastructural diversity analyzed in the research on renewable energy sources (RES). By providing a visual representation of the study regions, the map emphasizes the breadth of geographical conditions considered, which is essential for understanding the variations in attitudes and barriers toward RES adoption.

The selection of these countries reflects the differing levels of RES integration and energy policy frameworks present across Europe. Poland, with a lower share of RES in its energy mix, faces challenges related to high costs and limited infrastructure. Sweden, a leader in RES adoption, demonstrates advanced integration of renewable technologies, while France provides a unique perspective with its significant reliance on nuclear energy but moderate use of RES, primarily wind and solar. These differences provide a comprehensive context for understanding how national energy frameworks influence societal attitudes toward renewable energy.

The study builds upon this geographical and infrastructural diversity to examine perceptions, knowledge, and barriers to RES adoption. The inclusion of data from these distinct regions allows for a comparative analysis, enabling the identification of both universal trends and country-specific challenges in the transition to renewable energy. The findings contribute to a broader understanding of the factors influencing public acceptance of RES and the barriers that must be addressed to promote their widespread adoption.

Despite these efforts, the study acknowledges some limitations. Reliance on online distribution channels may have introduced biases because participation required digital access, potentially excluding certain demographic groups. Additionally, the geographic scope was limited to three countries, limiting the generalizability of the findings. These limitations suggest that future studies should expand the geographic scope and adopt random sampling methods to increase representativeness and inclusiveness.

Three nations with varying degrees of RES use were chosen, with the assumption that adult residents would participate in the study. Countries expected that a survey would be representative if a comparable number of people participated in it and that surveys would be correctly, despite the fact that each country populations varied greatly. The authors had little influence on the sample’s shape because it was an online poll that merely required the participation of respondents.

The three sections of the study were Environmental Responsibility (A), Knowledge and Use of Renewable Energy (B), and Barriers (C). These three sections each comprised five statements. If a respondent agrees with the statement, they were asked to mark their response. The evaluation comments were graded on a five-point Likert scale, with 1 denoting complete disagreement and 5 denoting complete agreement. In order to identify the most challenging obstacles to overcome, a series of statements were made that revealed society’s limitations in encouraging the development of hydrogen energy. The following were the survey’s statements:

A. Being accountable for the environment, my town or village, and the surrounding landscape.
  A1. I feel accountable for the energy sources I choose to utilise on my farm, as they have an impact on the surroundings.
  A2. My decisions and actions have an impact on my surroundings.
  A3. Citizens should avoid ecological catastrophes because of RES.
  A4. A developed society places a high priority on the usage of renewable energy sources.
  A5. Citizens now have access to cutting-edge technologies that are good for the environment.
B. Understanding of RES and their application.
  B1. Clean energy comes from renewable sources.
  B2. Using RES produces no carbon dioxide.
  B3. RES are used more willingly when people are aware of them.
  B4. Humans can safely use renewable energy.
  B5. The utilisation sources of renewable energy benefits the environment.
C. Obstacles.
  C1. My country has renewable energy options, but they are highly expensive.
  C2. Installing and using RES effectively is expensive.
  C3. The RES infrastructure in my nation is underdeveloped.
  C4. The use of RES solutions is still challenging due to a knowledge gap and a shortage of installation and service firms.
  C5. My understanding of RES might use some improvement, and what little I do know comes from people I know.

High scores indicate that the responder is pro-environmental (part A), knows about renewable energy or utilises it (part B), and is aware of the issues surrounding renewable energy (part C).

In order to assess the makeup of the study’s participants, the survey also included a section to gather data on basic responder characteristics.

The survey findings could be analysed in terms of the ‘reliability’ of the response thanks to the use of a five-point Likert scale. standardisation and Cronbach Alpha For this study, tests for Cronbach Alpha were utilised for every set of questions as well as for all surveys. The findings of the analysis were then interpreted in accordance with Hair et al. [127]. When the Cronbach’s alpha value is greater than 0.7, it is presumed that the data acquired are suitable for further study. It was determined which ratings were most frequently provided by the respondents by a scale analysis. The evaluation’s findings were then examined and calculated, including basic statistics and percentages of each evaluation.

## 4. Results

Finding opportunities for improvement is a crucial part of planning and carrying out new initiatives, as the literature demonstrates. The attainment of long-term goals based on cutting-edge analyses is supported by a variety of management techniques [8,53,130]. The writers made an effort to pinpoint the obstacle that has to be removed for society to see new energy technology favourably.

Questionnaire responses were pre-verified. 12 428 people in total answered the survey’s questions. Since every questionnaire was correctly filled out in its entirety, more analysis of the surveys was necessary (Table 2).

**Table 2:** Surveys included throughout the investigation (personal study).

Respondents	Quantity	Percentage Fraction
Number of respondents, including:	12 428	100%
correct	12 428	100%
rejected	0	0%

Initially the respondents’ organisational structure was examined (Table 3). Individual answer percentages were computed. Although the responders’ organisational structure was different, several elements were repeated. Most likely, the survey’s low completion rate was caused by the survey’s challenging subject. This also suggests a lack of awareness and expertise, though.

**Table 3:** Criteria of the responders (personal research).

Feature	Answer	Percentage
Age	under 20 years old	0%
	21–30 years	11%
	31–40 years	37%
	41–50 years	41,9%
	51–60 years	7,9%
	over 60 years old	2,5%
Gender	male	58%
	female	42%
Country	Poland	32%
	Sweden	34%
	France	34%
Education	primary education	0
	secondary education	27%
	higher education	73%
Residence	village	18,30%
	city up to 50k residents	9,70%
	city up to 100k residents	11,30%
	city up to 200k residents	12,60%
	city of 200k – 400k residents	22%
	city over 500k residents	26%
using any RES solution	yes	100%
	no	0%

The structure was balanced when the respondents’ genders were taken into consideration, with a majority of men. In order to maintain the balance of the nations from which the responses came, respondents filled out an Internet form that counted the numbers from various countries. Participants in the study ranged in age from 19 to 70, with the average age between 41 and 50. The requirement for participation in the study was that all respondents must be engaged in their profession. Test participants with secondary or higher education were included, which may be relevant to the topic of the questionnaire. The majority of the responders were from major cities with populations greater than 500,000.

Analysis was done on the main study’s findings. First, the total number of ratings for each statement was determined. These data were used to create a study of the association between individual summaries, accounting for the ratings given (Table 4). The following stage was calculating the standardised Cronbach’s alpha using the determined correlations.

**Table 4:** Individual question correlation matrix (personal study).

	A1	A2	A3	A4	A5	B1	B2	B3	B4	B5	C1	C2	C3	C4	C5
A1	1.00	–	–	–	–	–	–	–	–	–	–	–	–	–	–
A2	0.92	1.00	–	–	–	–	–	–	–	–	–	–	–	–	–
A3	0.76	0.72	1.00	–	–	–	–	–	–	–	–	–	–	–	–
A4	0.71	0.65	0.61	1.00	–	–	–	–	–	–	–	–	–	–	–
A5	0.67	0.59	0.56	0.50	1.00	–	–	–	–	–	–	–	–	–	–
B1	0.39	0.52	0.38	0.22	0.13	1.00	–	–	–	–	–	–	–	–	–
B2	0.40	0.29	0.41	0.28	0.11	0.59	1.00	–	–	–	–	–	–	–	–
B3	0.36	0.43	0.33	0.26	0.21	0.31	0.05	1.00	–	–	–	–	–	–	–
B4	0.29	0.31	0.44	0.11	0.25	0.19	0.37	0.22	1.00	–	–	–	–	–	–
B5	0.12	0.24	0.19	0.25	0.37	0.28	0.02	0.49	0.61	1.00	–	–	–	–	–
C1	0.25	0.33	0.51	0.28	0.03	0.17	−0.10	0.27	0.53	0.17	1.00	–	–	–	–
C2	0.29	0.31	0.44	0.16	0.23	0.02	0.34	0.15	0.09	0.03	0.14	1.00	–	–	–
C3	0.08	0.21	0.30	−0.09	0.25	0.08	0.25	0.09	0.27	0.34	0.09	0.01	1.00	–	–
C4	−0.02	0.08	0.12	−0.03	0.05	0.13	0.02	−0.33	0.27	0.20	−0.13	0.39	0.18	1.00	–
C5	−0.17	−0.22	−0.19	0.12	−0.08	0.42	−0.29	−0.15	−0.34	−0.28	−0.16	−0.14	−0.40	−0.31	1.00

The analysis of the data in Table 4 highlights diverse societal attitudes towards renewable energy sources (RES), reflecting both environmental awareness and barriers that hinder their adoption. Group A, focused on environmental responsibility, received the highest ratings, indicating a high level of ecological awareness among respondents. Most participants felt responsible for their choice of energy sources and recognized their impact on environmental protection, demonstrating a societal understanding of the need to counteract ecological disasters through the use of RES. However, the analysis of Group B, which focused on knowledge about RES, revealed a moderate level of awareness, characterized by a neutral stance towards statements such as “Using RES does not emit carbon dioxide.” These findings suggest that while the public has a general understanding of the benefits of RES, concerns regarding their safety and accessibility persist. Group C, dedicated to barriers in using RES, revealed key obstacles. Respondents frequently pointed to high installation costs and a lack of sufficient financial incentives as primary issues limiting their ability to adopt these technologies. Similarly, concerns about underdeveloped RES infrastructure and insufficient technical knowledge were significant barriers identified by participants. These results demonstrate that economic and educational constraints are the main factors hindering the widespread adoption of renewable energy technologies. In conclusion, despite a strong sense of environmental responsibility among respondents, insufficient knowledge, economic barriers, and limited access to RES infrastructure significantly impede their widespread application. The findings highlight the need to increase public awareness through educational campaigns, financial support programs, and more accessible and comprehensible information about RES. This approach can help overcome both mental and practical barriers, thereby accelerating the energy transition towards sustainable development.

Scale statistics were used to begin the examination of the results. The variance, mean and standard deviation of a scale made up of all five examined items are shown in Table 5. It should be mentioned that the adopted scale accepts values for all items ranging from 1 to 75. Average 53,2187 on the scale, or almost 2/3 of the total, seems to be relatively high and reflects respondents’ positive attitudes towards environmental preservation and RES. The Cronbach Alpha coefficients for the survey sections indicate a high reliability of the questionnaire, with values exceeding 0.8 for most parts. This suggests that the survey items within each section were consistent in measuring the intended constructs. Specifically, the section “Being accountable for the environment” achieved the highest standardized Cronbach Alpha of 0.906, reflecting the strong coherence of statements addressing environmental responsibility. The section on “Understanding of RES and their application” also demonstrated high reliability (standardized Cronbach Alpha =  0.850), which supports the validity of the responses related to knowledge and awareness of renewable energy sources. Meanwhile, the section on “Obstacles” had the lowest, yet acceptable, reliability score (standardized Cronbach Alpha =  0.807). This could indicate greater variability in how respondents perceive barriers to adopting renewable energy. Scale statistics show an overall mean response score of 53.2187, which is relatively high compared to the scale’s possible range, reflecting a positive overall attitude towards renewable energy and environmental preservation. However, significant standard deviations within the sections highlight the diversity of opinions and awareness among respondents, especially in questions addressing barriers and specific technical knowledge. In conclusion, while the survey results demonstrate strong awareness and accountability regarding environmental issues and renewable energy, notable gaps persist in understanding and addressing barriers, particularly economic and infrastructure-related challenges. These findings emphasize the need for targeted interventions, such as educational campaigns and financial incentives, to enhance public knowledge and support for renewable energy adoption.

**Table 5:** Cronbach Alpha coefficients.

Part of the Survey	Cronbach Alpha	Standardized Cronbach Alpha
The complete questionnaire	0,849	0,865
A Being accountable for the environment, my town or village, and the surrounding land-scape	0,892	0,906
B Understanding of RES and their application.	0,835	0,850
C Obstacles	0,791	0,807

The analysis of the data presented in Table 6 indicates a generally positive attitude among respondents toward renewable energy sources (RES) and environmental preservation. The mean score of 54.11 reflects an overall favorable disposition, suggesting that participants recognize the importance of sustainable practices and the role of renewable energy in achieving them. However, the standard deviation of 8.90 reveals some variability in the responses, indicating differences in knowledge levels, awareness, or opinions across the surveyed population. The variance of 95.31 further supports this observation, showing a moderate level of diversity in attitudes within the respondent group. These findings highlight the reliability of the survey data and the statistical consistency of the results, providing a solid foundation for drawing conclusions about public perceptions of RES. Despite the generally positive outlook, the observed variability points to the need for targeted efforts to address existing gaps in knowledge and understanding. Educational campaigns, public outreach initiatives, and accessible resources about the benefits and practical applications of renewable energy could help bridge these gaps and foster a more uniform level of awareness and acceptance among the population. By focusing on these areas, policymakers and stakeholders can build on the existing positive attitudes while addressing uncertainties or misconceptions, ultimately encouraging broader adoption and support for renewable energy solutions. The results of each question’s score singly and for each group of topics were then calculated, together with the mean and standard deviation (Table 7).

**Table 6:** The scale statistics.

Mean	Variance	Standard Deviation
54,10583	95,31054	8,89672

**Table 7:** Statistics for topics and specific issue groupings.

Question	Mean	Standard Deviation
A	4,21	1,35
A1	3,86	1,19
A2	3,79	1,23
A3	3,61	1,36
A4	4,08	1,18
A5	4,12	0,72
B	3,89	1,24
B1	3,75	0,98
B2	3,97	1,31
B3	3,81	1,22
B4	3,69	1,19
B5	2,42	1,25
C	3,48	1,17
C1	2,57	1,26
C2	3,05	0,83
C3	3,11	0,97
C4	2,84	1,05
C5	2,69	0,71

According to a study of the data for each group of statements, group A received the highest ratings, meaning that the respondents agreed with the majority of the analysed sentences. It is a collection of phrases that have to do with environmental protection and are frequently debated in public on forums, in the news, and on social media. Additionally, “going green” has recently gained in popularity, therefore the results of this group (A) are not particularly shocking. This collection of statements also had the highest standard deviation, indicating the widest range of evaluations across the group.

Group C’s average was the lowest, indicating that the respondents either disagree with or don’t care about the assertions. This group focuses on RES usage barriers and their root causes. The lowest findings in this group suggest that there is little public understanding and awareness of the issue, and that people are not interested in the advantages. This demonstrates the need for substantial support for the growth and development of RES through initiatives that advance not only ecological but also, and most importantly, technology education for the general population. It should be highlighted that society wants clean, intelligible data and that information is widely accessible (which is also a technology factor). The public’s perception of safety and accessibility can be improved with the use of this data.

Respondents use renewable energy sources because they are aware of their responsibility to protect the environment. This indicates that society still has a mental hurdle to clear regarding the question of ecological sustainability. It may be concluded from an analysis of the other assertions in group A that the respondents agreed with them, albeit with a partial shift to indifference.

The average response in group B for question B5 (The use of renewable energy sources benefits the environment.) was 2.42, indicating that the respondents were unsure whether to choose the response of indifference or disagreement. The replies in the remaining cases in this group showed swings between apathy and agreement with a statement. This finding shows that society is aware of the advantages of adopting RES, yet the man still worries about his safety. Response variations could also be the result of a conflicting concept of security; in this case, security could relate to both the safety of technical conditions and the security of access to the energy source.

With an average question score of 2.57, Statement C1 (My country offers renewable energy sources, but they are very expensive) was scored the poorest. This means that respondents did not agree with this statement, often not at all.

The reactions to the other claims in this group, however, ranged from disagreement to empathy. If the respondents believe that the nation lacks qualified advisors, technological infrastructure, and understanding of RES and its potential, then this is to be expected.

Percentage values of the ratings given to individual statements were also computed in order to study the response structure. According to the analysis of grades 4, which is cases in which respondents completely agree with a given statement, the highest percentages of this assessment were recorded for statements A1 (I feel responsible for the energy sources I choose to use on my farm, as they have an impact on the environment) and A3 (People should avoid ecological catastrophes because of RES). This shows that there is a great opportunity for society to adopt renewable energy technologies. The majority of people gave it a score of 4 (agree). People today have access to cutting-edge environmental technologies; however, it is unclear whether this is true in practise. The public may have access to these technologies but may not always be able to use them. Accessibility in this sector may be significantly impacted by an economic barrier, such as a lack of financial resources or state assistance in the form of subsidies, discounts, or financial incentives. The majority of the statements in category B received a grade of 3 (neutral; neither yes nor no). The respondents’ lack of interest suggests that they are unaware of the technology involved in generating energy from hydrogen as well as the advantages and safety of doing so. This supports earlier findings that society needs precise information on renewable energy technologies. No interesting findings can be drawn from the analysis of the rating of 2 (disagree). The most prevalent response was 1 (totally disagree) for statement C3 (My country’s RES infrastructure is underdeveloped). The one with the lowest score is this one. Conclusion: The respondents vehemently disagree with this assertion, and the absence of their confirmation does not indicate a genuine lack of infrastructure, but rather the respondents’ lack of interest in the topic, which has caused them to lack knowledge in this area. Residents’ lack of information about renewable energy is the reason why respondents are wary of using it. This is made worse by people’s generalised aversion to novelty and the uncharted.

Therefore, as depicted in Fig 1, it is important to enhance public awareness of this issue by various social campaigns, television programmes, and press articles that highlight the advantages of adopting green energy, particularly RES. Of course, persuading the entire population is impossible. However, overcoming obstacles to the use of renewable energy can be done by persuading some of society.

## 6. Discussion

Even in nations where modern energy technologies are not widely used, society is encouraged to adopt reforms that will safeguard the environment since there is a high level of awareness of the ongoing degradation of the environment.

The respondents are not persuaded that the energy derived from renewable energy sources has an adequate level of security, where security can be regarded as access stability and technical stability (i.e., availability). They also possess unproven expertise on the subject, and there aren’t many experts in this area. Those who are naked have the opinion that renewable energy sources are expensive and hence unaffordable, lack infrastructure, and offer no substantial economic advantages. As a result, there has been little preparedness for the intensive development of RES in countries.

The study’s objective is to stimulate participants’ interest in RES. Participating in a survey, according to the authors, might nudge respondents to hunt for more data on this subject. For the writers, but more significantly for the environment, it would be seen as a great accomplishment. In order to strengthen the relevant case study and perform a more extensive analysis of the data from multiple aspects, we also want to use new statistical approaches, which will increase the quality, accuracy, and validity of our conclusions.

The main scientific value of the paper lies in its comprehensive exploration of societal perceptions, attitudes, and barriers related to renewable energy sources (RES). The study offers valuable insights into the environmental awareness of participants, even in regions where modern energy technologies are not widely adopted. It identifies key concerns regarding the security, affordability, and lack of preparedness for the intensive development of RES. The analysis of knowledge gaps and discrepancies in perceptions emphasizes the importance of educational initiatives and public awareness campaigns. The paper contributes to the scientific understanding of factors influencing the acceptance and adoption of RES, paving the way for targeted interventions to promote sustainable energy practices. Additionally, the proposed use of new statistical approaches enhances the quality, accuracy, and validity of the study’s conclusions, further strengthening its scientific value.

The study conducted in Poland, Sweden, and France reveals a multifaceted landscape of public attitudes, knowledge levels, and barriers to the adoption of renewable energy sources (RES). Respondents across all three countries exhibit a strong sense of environmental accountability, suggesting heightened awareness of individual and collective impacts on ecological sustainability. This finding aligns with the Environmental Responsibility Theory, which emphasizes that individuals perceive their actions as integral to achieving ecological balance. In Poland, respondents demonstrated a particularly high level of environmental awareness, as indicated by an average rating of 4.21 on a five-point scale. Despite this consciousness, significant concerns persist regarding the security and affordability of renewable energy. These concerns encompass access stability, technical reliability, and the perceived high costs associated with RES. Such findings underscore the need for targeted initiatives that address these apprehensions and highlight the economic and environmental benefits of renewable energy. Studies, such as those by Latosińska and Miłek, have further emphasized the influence of education on shaping perceptions and self-assessments related to renewable energy, revealing a direct correlation between educational attainment and positive attitudes toward RES. In Sweden, respondents expressed a generally positive inclination toward renewable energy, with an average score of 3.89, reflecting favorable attitudes toward its benefits. However, notable knowledge gaps emerged as a barrier to broader acceptance, emphasizing the critical role of education and information dissemination. These results align with studies conducted by Kiesecker and Yang, which highlight the importance of addressing public misconceptions and enhancing awareness to facilitate the adoption of clean energy solutions. Similarly, in France, respondents demonstrated a strong sense of environmental responsibility and a willingness to engage in reforms aimed at environmental protection. Nevertheless, challenges related to high costs, underdeveloped infrastructure, and knowledge gaps were prominent. Such barriers not only hinder the adoption of RES but also necessitate interventions, such as regulatory reforms, financial incentives, and public awareness campaigns, to overcome these challenges effectively. Research by Piacentini and Rossetto corroborates these findings, emphasizing the critical need for policies that support the transition to sustainable energy practices.

A comparative analysis of the three countries highlights unique challenges and opportunities. Poland’s reliance on coal poses significant economic and cultural obstacles to transitioning to RES, despite strong environmental awareness. Conversely, Sweden’s leadership in renewable energy adoption showcases the potential of well-funded initiatives and widespread public advocacy. However, local resistance to specific projects, such as wind farms, underscores the complexity of achieving consensus, even in progressive contexts. France’s energy landscape, characterized by the dominance of nuclear power, presents distinct challenges. While nuclear energy provides low electricity costs and stable supply, it also creates a psychological and structural barrier to the adoption of renewable technologies. These observations align with the Diffusion of Innovation Theory, which posits that socio-economic and cultural contexts significantly influence the rate and success of technology adoption. The study also delves into the understanding and application of RES, revealing a generally positive attitude toward clean energy sources but highlighting discrepancies in knowledge levels. For instance, the lower agreement with the statement “The use of renewable energy sources benefits the environment” reflects uncertainty or ambivalence among respondents regarding the environmental advantages of RES. This finding aligns with the Technology Acceptance Model, which suggests that perceived ease of use and usefulness are critical to technology adoption. Addressing these gaps through tailored educational efforts can significantly enhance public understanding and acceptance of renewable energy technologies. Barriers to RES adoption, particularly those related to economic and infrastructural challenges, remain a recurring theme across all three countries. High installation costs, underdeveloped infrastructure, and limited technical knowledge were frequently cited by respondents. For example, the statement “My country has renewable energy options, but they are highly expensive” received the lowest average score, reflecting widespread concerns about affordability. These findings align with existing literature emphasizing the importance of financial incentives, subsidies, and infrastructure investments in overcoming adoption barriers. Studies by Burke and Stephens and Inglesi-Lotz highlight the pivotal role of economic support and transparent communication in fostering public trust and accelerating the energy transition. In conclusion, the study’s findings emphasize the interplay between environmental awareness, knowledge gaps, and structural barriers in shaping public attitudes toward RES. While respondents across Poland, Sweden, and France exhibit a positive disposition toward renewable energy, challenges such as perceived high costs, underdeveloped infrastructure, and limited technical knowledge require targeted interventions. Addressing these barriers through educational campaigns, financial support programs, and infrastructure development can foster greater public acceptance and utilization of RES. By integrating these findings with theoretical frameworks and existing literature, this research provides a comprehensive understanding of the factors influencing renewable energy adoption and offers actionable insights to guide future policy and educational initiatives. Understanding and applying renewable energy sources (RES) is critical to fostering a sustainable energy transition, and the study reveals both positive inclinations and significant concerns among respondents. Part B delves into the understanding and application of RES, revealing a generally positive inclination towards clean energy sources. The average score for this section was 3.89, indicating a favorable disposition towards the benefits of renewable energy. Notably, Statement B5 (“The use of renewable energy sources benefits the environment”) received a lower score of 2.42, suggesting uncertainty or ambivalence among respondents regarding the environmental advantages of RES. The study presented in this paper also reveals a discrepancy in knowledge levels, with some participants lacking expertise in renewable energy sources. This knowledge gap is seen as a barrier to wider acceptance and highlights the importance of educational efforts to enhance public understanding. The low interest and disagreement observed in certain statements within the study’s obstacle category (Group C) underscore a need for substantial support for the growth and development of RES through technology education initiatives. The exploration of obstacles in Part C highlights significant concerns. Statement C1 (“My country has renewable energy options, but they are highly expensive”) received the lowest average score of 2.57, indicating a disagreement among respondents. However, other obstacles, such as the perceived high cost of installing and using RES (C2), underdeveloped RES infrastructure (C3), and a knowledge gap hindering the use of RES solutions (C4), reflect challenges that need to be addressed for widespread adoption. These findings align closely with the demographic analysis, which reveals that education levels and residential locations significantly influence perceptions of RES. Respondents with higher educational backgrounds demonstrated greater awareness and understanding of renewable energy benefits, while those in urban areas were more likely to recognize infrastructure challenges. This demographic diversity underscores the need for tailored educational and infrastructural interventions that address the specific needs and concerns of varied population groups, ultimately enhancing public engagement with RES solutions. The demographic analysis revealed a diverse participant profile, with respondents spanning various age groups, genders, educational backgrounds, and residence types. Notably, 100% of the respondents reported using some form of RES solutions, showcasing a sample actively engaged in renewable energy practices. The statistical analysis, including Cronbach Alpha coefficients, indicates high reliability and internal consistency of the survey instrument. The overall Cronbach Alpha for the complete questionnaire was 0.849, suggesting that the data collected are suitable for further study. The breakdown of Cronbach Alpha coefficients for each section (A, B, and C) further supports the reliability of the responses. In terms of the scale statistics, the mean score for the entire scale was 51.06, with a variance of 96.28 and a standard deviation of 8.90. This reflects a relatively high and consistent positive attitude towards environmental preservation and RES, as the average score is almost two-thirds of the total scale. Analysing responses for individual statements within each section, the study identified nuances in public perceptions. Group A, focusing on environmental accountability, received the highest ratings, indicating agreement with statements related to environmental protection. In contrast, Group C, addressing obstacles, received the lowest average score, suggesting a lack of interest or awareness regarding barriers to RES adoption. The correlation matrix and Cronbach Alpha coefficients provide additional insights into the relationships between survey items and the internal consistency of each section. The standardized Cronbach Alpha values indicate high reliability, supporting the robustness of the survey instrument. The respondents’ perceptions of renewable energy infrastructure also vary, with some expressing skepticism about the underdevelopment of RES infrastructure in their countries. This discrepancy could be attributed to a lack of awareness and interest in the topic, emphasizing the importance of public education and awareness campaigns. The discussion underscores the significance of addressing these barriers through various channels, including social campaigns, television programs, and press articles, to enhance public awareness of the advantages of adopting renewable energy, particularly RES. It acknowledges the challenges of persuading the entire population but emphasizes the potential impact of convincing a significant portion of society.

These findings align closely with the demographic analysis, which reveals that education levels and residential locations significantly influence perceptions of RES. Respondents with higher educational backgrounds demonstrated greater awareness and understanding of renewable energy benefits, while those in urban areas were more likely to recognize infrastructure challenges. This demographic diversity underscores the need for tailored educational and infrastructural interventions that address the specific needs and concerns of varied population groups, ultimately enhancing public engagement with RES solutions. The study’s results, particularly in Part A, align with the Environmental Responsibility Theory [66–68], which suggests that individuals feel a sense of accountability for their impact on the environment. The respondents expressed a strong sense of responsibility for their energy choices, emphasizing the importance of using renewable energy sources to mitigate environmental consequences. This theory [69–70] helps explain the high ratings in statements related to environmental accountability (A1, A2, A3, A4, A5). The Technology Acceptance Model [71–74] provides a lens through which to interpret the findings in Part B, focusing on the understanding and application of RES [75–76]. The overall positive inclination towards clean energy sources (B) suggests a general acceptance of renewable technologies. However, the lower score for B5 (“The use of renewable energy sources benefits the environment”) indicates uncertainty or ambivalence, which can be attributed to concerns about the perceived advantages or lack of awareness. The TAM emphasizes perceived ease of use and perceived usefulness as crucial factors in technology adoption, and this is reflected in the respondents’

attitudes [77–80]. The Diffusion of Innovation Theory [81–86] can also be applied to understand the varying attitudes towards RES. The study identifies a diverse set of responses, with some participants showing a high level of awareness and positive attitudes, while others exhibit skepticism or lack of interest. This diversity can be explained by the theory, which posits that innovations are adopted at different rates based on individuals’

characteristics, including their awareness, risk tolerance, and socio-economic status. The study’s results suggest a positive trend in societal attitudes towards environmental responsibility and the adoption of renewable energy. However, challenges such as perceived high costs, underdeveloped infrastructure, and knowledge gaps need targeted interventions to facilitate broader acceptance and utilization of RES. The demographic diversity of respondents enhances the generalizability of the findings, offering valuable insights for policymakers, educators, and advocates seeking to promote sustainable energy practices.

## 6. Conclusion and policy implications

### 6.1. Key results

The research gap identified in the introduction was addressed through the survey conducted in this study, providing a valuable instrument to assess societal barriers stemming from respondents’ concerns and uncertainties regarding renewable energy sources (RES). This aligns with previous studies highlighting that public misconceptions and limited knowledge often act as significant barriers to RES adoption [74,129]. The findings contribute to understanding how societal perceptions influence the acceptance of renewable technologies, reinforcing the importance of bridging knowledge gaps through targeted educational efforts [37,131]. One of the key findings of this study is the significant role of environmental responsibility in shaping public attitudes toward RES. Respondents demonstrated a strong sense of accountability for their environmental impact, consistent with the Environmental Responsibility Theory [132,133]. This is supported by prior research indicating that heightened environmental awareness is a driving factor in promoting sustainable behaviors and renewable energy adoption [129]. The study also highlights the influence of demographic factors, such as education levels and urban versus rural residency, on public perceptions of RES. These findings are corroborated by studies showing that higher education levels are positively correlated with greater awareness and acceptance of renewable technologies [134,135]. Another significant insight is the impact of economic and infrastructural barriers on RES adoption. Respondents frequently cited high installation costs and underdeveloped infrastructure as critical challenges, aligning with global trends documented in the literature [4,136]. The study’s results emphasize the importance of policy interventions, such as financial incentives and subsidies, to alleviate these economic concerns and promote widespread adoption. The survey revealed significant findings. Respondents consistently demonstrated a strong sense of environmental responsibility, aligning with the Environmental Responsibility Theory [7]. This is reflected in heightened accountability for personal environmental impact, which parallels studies showing environmental awareness as a driver of sustainable behavior [129]. Additionally, demographic factors, such as education levels and rural versus urban residency, were shown to significantly influence perceptions. Higher education levels correlated with greater understanding and acceptance of RES, supporting findings from [134,135].

This aligns with findings by González-Ramos et al. [137], who identified targeted financial support as a key enabler in overcoming adoption barriers. The sociological approach of this study offers additional value by emphasizing the interplay between societal attitudes and technical knowledge gaps. The findings reveal that while respondents generally favor renewable energy, significant discrepancies in technical understanding persist, particularly regarding the environmental benefits of RES. Similar gaps have been documented in studies by Yang et al. [138] and Piwowar & Dzikuć [111], highlighting the need for comprehensive educational campaigns to improve public understanding of RES technologies and their advantages. Moreover, the study contributes to ongoing discussions about the importance of localized approaches to renewable energy adoption. Differences observed among respondents in Poland, Sweden, and France underscore the influence of cultural, economic, and policy contexts on public perceptions and acceptance. This aligns with the Diffusion of Innovation Theory [139], which emphasizes the role of socio-economic and cultural factors in shaping the adoption of new technologies. By focusing on these localized dynamics, this research complements prior studies advocating for context-specific strategies to enhance renewable energy deployment [140]. In conclusion, while the study’s methodological and geographical constraints limit its scope, it offers a strong foundation for future research. Expanding the study to include diverse demographic groups and additional countries would enhance its generalizability and provide a more comprehensive understanding of the factors influencing RES adoption. Building on the insights from this research and integrating findings from prior studies, such as those by Paul & Lim [34] and González-Ramos et al. [137], will be crucial for advancing the global transition to renewable energy. The results underscore the need for interdisciplinary approaches that combine sociological, economic, and technical perspectives to address the complex barriers to renewable energy adoption effectively.

The sociological lens of this study adds valuable context to discussions about RES adoption. It emphasizes the interplay between societal attitudes and technical knowledge, revealing significant gaps in public understanding of RES benefits. This finding aligns with Yuan et al. [138] and Wuni and Shen [141], who also stressed the importance of educational campaigns in fostering public acceptance. Localized approaches to RES adoption were identified as essential, with the study showing significant differences across Poland, Sweden, and France. These variations highlight the influence of cultural, economic, and policy contexts, supporting the Diffusion of Innovation Theory [139]. Prior research has similarly advocated for tailored strategies to address local dynamics [140].

While the study offers valuable insights, certain limitations must be acknowledged. The questionnaire was specifically designed for this research, introducing potential subjectivity, a limitation also noted in Żywiołek [42]. The reliance on non-random sampling methods may have affected respondent diversity, as observed in studies by Miafodzyeva & Brandt [135]. Geographical constraints further limit the generalizability of findings, underscoring the need for cross-national comparisons in future research [134]. Future studies should broaden the sample scope to include more diverse demographic groups and countries. Integrating insights from interdisciplinary perspectives—sociological, economic, and technical—will be crucial for addressing complex barriers to RES adoption. Building on this foundation and previous research [137] will help advance global efforts toward a sustainable energy future. This research emphasizes that addressing knowledge gaps, economic barriers, and infrastructural challenges requires coordinated educational initiatives, policy reforms, and localized interventions. By aligning these efforts with societal dynamics, it is possible to facilitate a more inclusive and effective transition to renewable energy sources.

### 6.2. Implications

The results have important policy implications for the dissemination of RES adoption in the rural areas of Poland, Sweden, and France and can also be extended to other similar contexts within the European Union. The demonstrated lack of public awareness and technical knowledge related to renewable energy calls for special educational campaigns. This also means that policy makers need to raise a loud and clear message, showing that information concerning the environmental benefits, cost-effectiveness, and practical applications of RES is more accessible and attractive. This will help dispel some knowledge gaps and misconceptions acting as barriers to its adoption.

The findings also show how financial incentives overcome economic barriers to the realization of RES. With such a prospect in sight, policy makers seriously ought to introduce or expand subsidies, tax benefits, and low-interest loan programs specifically with the aim of bringing down the initial costs for households and businesses in installing RES. This might handle the prevailing affordability concerns related to renewable energy technologies and get more investment towards sustainable solutions. This means that policy efforts in this period of time must aim at increasing energy grid capacity and reliability, in concert with increased access to better services and technologies associated with RES in rural areas. Indeed, upgrading the energy infrastructure-smart grids and storage-contributes greatly to improving the accessibility and stability of renewable energy systems.

The study has also indicated further that attitudes and perceptions vary with socio-economic and cultural contexts, hence policies have to be localised and culturally sensitive. Interventions have to be focused on challenges in varied regions, for example, Polish dependence on coal, local Swedish resistance to renewable energy projects, and the culturally deeply ingrained dependence of France on nuclear energy. These will have to be complemented by advocacy programs at the community level for trust and acceptance of the renewable technologies. Policymakers should also address the structural barriers of limited technical expertise and service availability. Programs for training and certification of professionals in the installation and maintenance of RES systems would improve the quality and availability of technical support. Furthermore, research and development into renewable energy technologies will further advance the technology and public confidence in the safety and reliability of the systems.

Results have pointed out attention for the presence of barriers on social and mental aspects-just as taken with technical and economic barriers. It therefore urges that embedding renewable energy education into schools-from curriculum or public programs highlighting personal roles within the energy transition process-should also be encouraged within the scope of work by governments and stakeholders. It instills a feeling of responsibility related to the natural environment and engages the general public with the switch-over process to renewable energy solutions quite effectively.

### 6.3. Limitations

The online survey methodology, while advantageous, presents certain limitations that may have influenced the data collection process. One significant challenge is the potential overrepresentation of demographic groups with easy internet access, which could lead to the underrepresentation of rural residents with limited connectivity. This discrepancy may distort the understanding of attitudes and knowledge about renewable energy sources (RES), particularly among groups facing the most substantial technological and infrastructural barriers. It is also important to note that the level of RES adoption varies significantly among the surveyed countries, which may have influenced respondents’ perceptions. For instance, Sweden, a leader in RES adoption, generates approximately 60% of its energy from renewable sources, primarily hydropower and biomass. In contrast, Poland’s RES share is around 16%, reflecting a heavier reliance on fossil fuels. France, while predominantly reliant on nuclear energy, has a RES share of about 25%, mainly from wind and solar energy. These differences likely shaped perceptions of accessibility, costs, and the benefits of RES in each country. Another limitation is the reliance on self-reported data, which is susceptible to subjective biases. Self-reported responses may reflect perceptions and intentions more than actual behaviors or knowledge. Such data is prone to cognitive biases, including confirmation bias or the desire to provide socially acceptable answers, potentially affecting the accuracy and objectivity of the results. To address these limitations, future studies should incorporate objective data to complement and validate survey findings. For example, including household energy consumption statistics could provide valuable insights into actual energy usage patterns and RES adoption. Additionally, analyses of local energy infrastructure could offer a deeper understanding of regional availability and development of RES, helping to identify barriers and opportunities more effectively.

### 6.4. Future research directions

Despite these limitations, the study provides valuable insights into societal perceptions of RES, but its findings should be interpreted with these constraints in mind. Expanding future research to include the elements mentioned above would allow for a more comprehensive analysis and enhance the practical applicability of the study’s results.

To build upon the findings of this study and address its limitations, several directions for future research are recommended. First, longitudinal studies should be conducted to track changes in attitudes, knowledge, and societal barriers regarding renewable energy sources (RES) over time. This approach would provide insights into the dynamics of public perceptions and the effectiveness of policy interventions, educational campaigns, and infrastructure developments. Tracking these changes longitudinally would allow researchers to identify trends and evaluate the long-term impacts of initiatives aimed at increasing RES adoption. Second, the application of mixed methods research is strongly encouraged to enhance the depth and breadth of understanding in this field. By combining quantitative survey approaches with qualitative methods, such as in-depth interviews or focus groups, researchers can capture a more nuanced view of societal attitudes and barriers. For example, interviews with residents of urban and rural areas could reveal contextual factors that influence perceptions of RES, such as local economic conditions, cultural attitudes, and the visibility of renewable energy infrastructure. This qualitative dimension would complement quantitative data, providing richer and more actionable insights. Third, future studies should expand the geographical scope to include a broader range of countries and regions with varying levels of energy infrastructure and policy development. The inclusion of nations with diverse economic, technological, and cultural contexts would improve the representativeness of the findings and facilitate cross-national comparisons. Such comparisons would be invaluable for identifying universal trends and context-specific challenges, thereby enhancing the global relevance and applicability of the research. By incorporating these approaches—longitudinal tracking, mixed methods, and expanded geographical coverage—future research can provide a more comprehensive understanding of the societal dynamics surrounding RES adoption. These methodologies will also help bridge knowledge gaps, inform targeted interventions, and support the development of effective policies to promote the transition to renewable energy systems on a global scale.
